# Prenylated flavonoid-enriched fraction from *Maclura tinctoria* shows biological activity against *Staphylococcus aureus* and protects *Galleria mellonella* larvae from bacterial infection

**DOI:** 10.1186/s12906-019-2600-y

**Published:** 2019-07-29

**Authors:** Ayla das Chagas Almeida, Lais Azevedo Rodrigues, Graziela dos Santos Paulino, Ananda Pereira Aguilar, Alisson Andrade Almeida, Sukarno Olavo Ferreira, Geraldo Célio Brandão, João Paulo Viana Leite, Andréa de Oliveira Barros Ribon

**Affiliations:** 1 Departamento de Bioquímica e Biologia Molecular, Universidade Federal de Viçosa, Viçosa, MG 36570-900 Brazil; 2 Departamento de Física, Universidade Federal de Viçosa, Viçosa, MG 36570-900 Brazil; 3 Escola de Farmácia, Departamento de Farmácia, Universidade Federal de Ouro Preto, Ouro Preto, MG 35400-000 Brazil

**Keywords:** *Maclura tinctoria*, Prenylated flavonoids, Antibacterial, *Galleria mellonella*

## Abstract

**Background:**

The Atlantic Forest biome extends along the entire Brazilian coast and is home to approximately 20,000 plant species, many of which are endemic; it is considered one of the hotspot regions of the planet. Several of these species are sources of natural products with biological activities that are still unknown. In this study, we evaluated the antimicrobial activity of 90 extracts derived from native Atlantic Forest tree species against *Staphylococcus aureus*, an important human and veterinary pathogen.

**Methods:**

Extracts from native Atlantic Forest tree species were evaluated for their antimicrobial activity against *S. aureus* by in vitro standard methods. Phytochemical fractionation of the extract from *Maclura tinctoria* was performed by liquid-liquid partitioning. LC-DAD-ESI-MS was used for identification of constituents in the most active fraction. Damage of cells and alterations in the permeability of cell membrane were determined by atomic force microscopy (AFM) and crystal violet uptake assay, respectively. In vivo antimicrobial activity was evaluated using *Galleria mellonella* larvae infected with *S. aureus* with survival data collected using the Kaplan-Meier method.

**Results:**

Among the organic or aqueous extracts tested here, 26 showed biological activity. Eight species showed relevant results, with a minimum inhibitory concentration (MIC) below 1 mg/mL. Antibacterial activity was registered for three species for the first time. An organic extract from *Maclura tinctoria* leaves showed the lowest MIC (0.08 mg/mL). Fractionation of this extract by liquid-liquid partitioning led to obtaining fraction 11FO d with a MIC of 0.04 mg/mL. This fraction showed strong activity against veterinary *S. aureus* isolates and contributed to the increased survival of *Galleria mellonella* larvae infected with *S. aureus* ATCC 29213. The bacterial surface was not altered by the presence of 11FO d, and no cell membrane damage was detected. The LC-DAD-ESI/MS analyses identified prenylated flavonoids as the major constituents responsible for the antibacterial activity of this active extract.

**Conclusion:**

A fraction enriched in prenylated isoflavones and flavanones from *M. tinctoria* showed in vitro and in vivo efficacy as antistaphylococcal agents. These findings justify the need for further research to elucidate the mechanisms of action of these compounds.

## Background

*Staphylococcus aureus* is responsible for high rates of morbidity and mortality worldwide and is the most common cause of skin infections, although it may also be associated with osteomyelitis, bacteremia and sepsis [1]. In cattle, it is the primary cause of mastitis, which is currently considered the main disease of dairy herds [2]. Great adaptations to different niches and hosts are determined in part by the variety of bacterial virulence factors that contribute to the establishment of the disease.

*S. aureus* has a great ability to develop antimicrobial resistance. The first penicillin-resistant strains arose in the mid-1940s, and as early as the 1960s, reports described the isolation of *S. aureus* with broad resistance to beta-lactams, especially methicillin (MRSA) [3]. At first, the infections caused by MRSA were limited to hospitalized patients (HA-MRSA), but since the 1990s, strains associated with community-acquired infections (CA-MRSA) have also been isolated [4]. There are different strains of CA-MRSA around the world that are typically prevalent in certain regions, such as the ST 80 strain that is disseminated throughout Europe and ST 30 in Australia [4]. The USA 300 strain, which is associated with infections in hospitals and in the community, was initially reported in the US. However, it has been isolated from different continents, and it has become a global concern due to its hypervirulence, reduced susceptibility to different antimicrobials and great epidemic potential.

Plant resources can also be sources of compounds with antimicrobial properties. Several studies on plant extract libraries have been established, and the results show the importance of this strategy for the discovery and development of new drugs [5, 6]. Coumarin derivatives have displayed activity against *S. aureus* and *Bacillus anthracis* DNA helicases [7]. Further reports have described the efficacy of intraperitoneal injections of a synthetic dicoumarin derivative in septicemic mice [8] and hypodermic injections of lysionotin in mouse pneumonia model [9], confirming the potential use of natural products as leads for new drugs.

The Atlantic Forest biome is the second-largest block of tropical forest in Brazil and has the second-highest biological diversity, behind only the Amazon. As an area of great potential for medical research, the forest originally covered 15% of the Brazilian territory but it presently suffers from constant deforestation pressure due to urban expansion and the withdrawal of trees for conversion into coal, which endangers the fauna and flora of the region. Considering the great diversity of the Atlantic Forest biome, which hosts plant species still unknown to science, along with the urgency to discover new antimicrobial agents, the present work presents a bioprospection of natural products in the native trees extracts from this ecosystem. The antimicrobial activity of some of these plants was demonstrated for the first time.

## Methods

### Microorganisms and culture conditions

*S. aureus* ATCC 29213 was used as a test strain to evaluate the antibacterial activity of the plant extracts. Four field isolates of *S. aureus* (302/4, 469/4, 4130, and 4651), which originated from cows presenting mastitis, were also used to determine the MIC of the active fraction. The bacteria were maintained on Brain Heart Infusion agar (BHA, Fluka, Munich, GER) or Müeller-Hinton agar (MHA, Himedia, Mumbai, IND). For the antibacterial assays, bacteria were streaked onto MHA and cultured overnight at 37 °C before being used for inoculum preparation as described below. Stock cultures were stored at − 80 °C in BHI medium containing 20% glycerol.

### Plant material and extraction

Plant material from 23 species (Table 1) native to the Atlantic Forest were collected from a forest fragment at the coordinates 20°48′07″ S and 42°51′31″ W, which is part of the Universidade Federal de Viçosa (UFV), Minas Gerais, Brazil. The permission issued by the Conselho Nacional de Desenvolvimento Científico e Tecnológico (CNPq) granted access to genetic heritage (permit No 010134/2014–0). A specimen voucher of all the samples was deposited in the VIC Herbarium of the UFV and given a voucher number (VIC 40.269). Identification was performed by Marcos Vinícius R.C. Simão with the aid of specialized bibliography, consultation with botanicals, and comparison with herborized material from the VIC Herbarium determined by specialists. The Brazilian Flora 2020 Project was used to check the species names and the botanical families. The leaves and stems were separated, dried in a ventilated oven at 40 °C for 48 h and then subjected to extraction by serial maceration using a dry vegetable/solvent ratio of 1:5 (w/v). Each sample was first extracted with a mixture of lower-polarity solvents (dichloromethane and methanol, 1: 1) followed by water, and for both extracts, the solvents were completely removed [10]. The tree species were enumerated randomly, and the extracts were identified by the abbreviations FO (organic extract of leaves), FA (aqueous extract of leaves), GO (organic extract of branches) and GA (aqueous extract of branches). Stocks of all the plant extracts were prepared at a 50 mg/mL concentration in dimethyl sulfoxide (DMSO, Vetec, Rio de Janeiro, BR) for use in the biological tests.

**Table 1:** Antimicrobial activity of plant extracts against *Staphylococcus aureus* ATCC 29213

Extract	Family	Species	Voucher number –VIC	Inhibition zone (mm)*	MIC (mg/mL)
2FO	Caesalpinioideae	*Senna macranthera*	40.533	0.0	–
2FA		*Senna macranthera*		0.0	–
2GO		*Senna macranthera*		0.0	–
2GA		*Senna macranthera*		0.0	–
4FO	Mimosoideae	*Anadenanthera peregrina*	40.213	10.0 ± 1.0	0.62
4FA		*Anadenanthera peregrina*		13.0 ± 1.0	0.31
4GO		*Anadenanthera peregrina*		9.0 ± 1,4	0.62
4GA		*Anadenanthera peregrina*		9.6 ± 0.6	0.62
6FO	Siparunaceae	*Siparuna guianensis*	40.409	0.0	–
6FA		*Siparuna guianensis*		0.0	–
6GO		*Siparuna guianensis*		0.0	–
6GA		*Siparuna guianensis*		7.0 ± 0.0	2.5
8FO	Rubiaceae	*Bathysa nicholsonii*	40.273	6.6 ± 1.1	10.0
8FA		*Bathysa nicholsonii*		6.3 ± 0.6	1.25
8GO		*Bathysa nicholsonii*		9.3 ± 0,6	0.62
8GA		*Bathysa nicholsonii*		0.0	–
9FO	Salicaceae	*Casearia sylvestris*	40.390	0,0	–
9FA		*Casearia sylvestris*		0.0	–
9GO		*Casearia sylvestris*		0.0	–
9GA		*Casearia sylvestris*		0.0	–
10FO	Moraceae	*Ficus eximia*	40.263	0.0	–
10FA		*Ficus eximia*		0.0	–
10GO		*Ficus eximia*		0.0	–
10GA		*Ficus eximia*		0.0	–
11FO	Moraceae	*Maclura tinctoria*	40.269	15.0 ± 1.0	0.08
11FA		*Maclura tinctoria*		10.0 ± 1.7	1.25
11GO		*Maclura tinctoria*		0.0	–
11GA		*Maclura tinctoria*		0.0	–
12FO	Sapindaceae	*Allophylus sericeus*	40.333	0.0	–
12FA		*Allophylus sericeus*		0.0	–
12GO		*Allophylus sericeus*		0.0	–
12GA		*Allophylus sericeus*		0.0	–
14FO	Meliaceae	*Trichilia pallida*	40.287	0.0	–
14FA		*Trichilia pallida*		0.0	–
14GO		*Trichilia pallida*		0.0	–
14GA		*Trichilia pallida*		0.0	–
16FO	Nyctaginaceae	*Guapira opposita*	40.227	0.0	–
16FA		*Guapira opposita*		0.0	–
16GO		*Guapira opposita*		0.0	–
16GA		*Guapira opposita*		0.0	–
18FO	Malvaceae	*Ceiba speciosa*	40.342	0.0	–
18FA		*Ceiba speciosa*		0.0	–
18GO		*Ceiba speciosa*		6.6 ± 0.6	1,25
18GA		*Ceiba speciosa*		0.0	–
22FO	Euphorbiaceae	*Alchornea glandulosa*	40.485	0.0	–
22FA		*Alchornea glandulosa*		7.3 ± 1.1	1.25
22GO		*Alchornea glandulosa*		0.0	–
22GA		*Alchornea glandulosa*		0.0	–
23FO	Annonaceae	*Annona sylvatica*	40.508	0.0	–
23FA		*Annona sylvatica*		0.0	–
23GO		*Annona sylvatica*		0.0	–
23GA		*Annona sylvatica*		0.0	–
24FO	Melastomataceae	*Miconia petropolitana*	40.397	19.0 ± 0.0	0.31
24FA		*Miconia petropolitana*		0.0	–
24GO		*Miconia petropolitana*		14.0 ± 1.73	0,31
24GA		*Miconia petropolitana*		0.0	–
28FO	Mimosoideae	*Piptadenia gonoacantha*	40.225	0.0	–
28FA		*Piptadenia gonoacantha*		10.0 ± 0.0	0.62
28GO		*Piptadenia gonoacantha*		8.0 ± 0.0	1.25
28GA		*Piptadenia gonoacantha*		8.3 ± 1.1	1.25
29FA	Apocynaceae	*Tabernaemontana hystrix*	40.611	8.6 ± 0.6	1.25
29GO		*Tabernaemontana hystrix*		12.6 ± 0.6	1.25
29GA		*Tabernaemontana hystrix*		0.0	–
30FO	Rubiaceae	*Psychotria vellosiana*	40.282	0.0	–
30FA		*Psychotria vellosiana*		0.0	–
30GO		*Psychotria vellosiana*		0.0	–
30GA		*Psychotria vellosiana*		0.0	–
32FO	Annonaceae	*Xylopia sericea*	40.432	0.0	–
32FA		*Xylopia sericea*		11.3 ± 0.6	0.31
32GO		*Xylopia sericea*		10.0 ± 0.0	0.62
32GA		*Xylopia sericea*		10.3 ± 0.6	0.31
34FO	Solanaceae	*Solanum cernuum*	40.323	0.0	–
34GO		*Solanum cernuum*		0.0	–
34GA		*Solanum cernuum*		0.0	–
40FO	Phytolaccaceae	*Seguieria langsdorffii*	40.202	0.0	–
40FA		*Seguieria langsdorffii*		0.0	–
40GO		*Seguieria langsdorffii*		0.0	–
40GA		*Seguieria langsdorffii*		0.0	–
44FO	Malvaceae	*Luehea grandiflora*	40.338	0.0	–
44FA		*Luehea grandiflora*		0.0	–
44GO		*Luehea grandiflora*		11.3 ± 0.6	0.62
44GA		*Luehea grandiflora*		0.0	–
47FO	Cercideae	*Bauhinia forficate*	40.549	0.0	–
47FA		*Bauhinia forficate*		0.0	–
47GO		*Bauhinia forficate*		0.0	–
47GA		*Bauhinia forficate*		0.0	–
51FO	Myrtaceae	*Myrciaria glazioviana*	40.584	0.0	–
51FA		*Myrciaria glazioviana*		11.3 ± 0.6	0.62
51GO		*Myrciaria glazioviana*		12.6 ± 0.6	0.62
51GA		*Myrciaria glazioviana*		10.5 ± 0.7	0.31
Ampicillin	*–*	*–*	–	28 ± 1.73	0.004

*FO* Organic extract of the leaves, *FA* Aqueous extract of the leaves, *GO* Organic extract of the branches, *GA* Aqueous extract of the branches, and *MIC* Minimum inhibitory concentration

*Mean zones of bacterial growth inhibition of 6 mm were considered significantly different (*p* < 0.0001) from the control (DMSO)

### Agar well diffusion assay

The antimicrobial activity was initially evaluated according to the well diffusion test. Inoculum was prepared as recommended by the Clinical & Laboratory Standards Institute (CLSI) [11]. A suspension of *S. aureus* ATCC 29213 corresponding to 0.5 McFarland was spread on MH agar medium. Holes of approximately 5 mm in diameter and 3 mm high were punched, and 20 μL of the extracts were added at a concentration of 50 mg/mL. DMSO and ampicillin (10 mg/mL) (A9518, Sigma-Aldrich, Dorset, UK) were used as controls. The plates were kept at 4 °C for 4 h and 37 °C for 24 h. The inhibition halos were measured in millimeters. The tests were performed on three independent biological replicates. The mean value ± SD for the replicates was calculated.

### Determining the minimum inhibitory concentration (MIC) of the plant extracts

A 96-well microplate was filled with MH broth, with extract concentrations ranging from 0 to 10 mg/mL and 10^5^ CFU/mL of a suspension of *S. aureus* 291213. The control wells consisted of inocula added to 100 μL of the MH broth and MH broth plus DMSO in the volume corresponding to the highest concentration of tested extract. The microplates were incubated at 37 °C for 24 h. Subsequently, 5 μL of violet *p*-iodonitrotetrazolium (INT, I8377, Sigma-Aldrich, Dorset, UK) was added at 2 mg/mL. After 2 h of incubation, the MIC was defined as the lowest concentration of extract for which the purple color formation, which was indicative of cell viability, was not observed. The MIC of ampicillin for *S. aureus* 29213 was also determined. The MIC of the active fraction was estimated as described above using the reference strain and the isolates *S. aureus* 302/4, 469/4, 4130, and 4651. The assays were performed using three biological replicates.

### Fractionation and phytochemical prospecting of the bioactive extract

The extract with the lowest MIC according to the preliminary screening was chosen for further assays. The phytochemical composition was investigated by liquid-liquid partitioning in which the bioactive extract (1.0 g) was resuspended in water (w) and partitioned into a separatory funnel in hexane (h), dichloromethane (d), ethyl acetate (ac), and n-butanol (b). A volume of 100 mL of each solvent was used in this step. The solvents were evaporated to give the 11 FO h (0.087 g), 11 FO d (0.333 g), 11 FO ac (0.017 g), 11 FO b (0.092 g), and 11 FO w (0.318 g) fractions. All the fractions were subjected to phytochemical analysis and an antibacterial activity assay. A phytochemical screening was performed by thin layer chromatography in glass plates covered with silica gel (Whatman, Kent, UK). The extract and fractions were investigated for the presence of cumarins, tannins, flavonoids, anthraquinones, essential oils, saponins, alkaloids, and triterpenes/steroids using standards and specific reagents for each class of secondary metabolites [12].

### LC-DAD-ESI/MS analyses

The most active fraction of the fractionated extract was subjected to analyses by LC-DAD-ESI/MS using UPLC (ACQUITY) system coupled with a photodiode array detector (Waters) and on Triple Quadrupole instrument (Waters ACQUITY® TQD) with an electrospray source (ESI) operating in the positive and negative mode. The ESI-MS/MS operation was performed using capillary voltage, 3500 V; capillary temperature, 320 °C; source voltage, 5 kV; vaporizer temperature, 320 °C; corona needle current, 5 mA; and sheath gas, nitrogen, 27 psi with argon as the collision gas, and the collision energy was set at 30 eV. Analyses were run in the full scan mode (100–1500 Da). Chromatographic separation was done on Acquity UPLC HSS C_18_ column (50 mm × 2.1 mm i.d.; 1.7 μm; Waters, USA) in combination with an Acquity UPLC guard column (5 × 2.1 mm; 1.7 μm; Waters, USA) [13]. The mobile phase consisted of water 0.1% formic acid (solvent A) and acetonitrile 0.1% formic acid (solvent B). The elution protocol was 0–11 min, linear gradient from 5 to 95% B. The flow rate was 0.3 mL min − 1, and the sample injection volume was 4.0 μL. The UV spectra were registered from 190 to 450 nm. Phenolic compounds were identified on the basis of the typical UV absorption for each phenolic class analyzed here, in addition to the typical fragmentation patterns obtained by MS/MS in comparison to the literature data [14–19].

### Effect of the 11FO d fraction on *S. aureus* growth

The reference strain *S. aureus* 29213 was used to analyze the antibacterial activity of the 11FO d fraction [20]. A bacterial growth curve with an initial inoculum of 10^6^ CFU/mL was created at different MIC values, in the absence and presence of the 11FO d fraction. The aliquots were recovered at specific times, serially diluted and plated on BHI to determine the CFU/mL. The experiments were performed using two biological replicates.

### Atomic force microscopy (AFM)

*S. aureus* ATCC 29213 was grown in BHI medium at 37 °C for 12 h, centrifuged (2100 x g for 15 min), washed three times in phosphate buffer (5 mM, pH 6.5) and resuspended in the same buffer at approximately 10^8^ CFU/mL. The plant extract was then added at the MIC (0.04 mg/mL) for 4 h at 37 °C. Cells grown in culture media without the fraction were used as the control. After centrifugation, the pelleted cells were deposited on mica substrates for AFM (Ntegra Prima, NT-MDT, RUS). The AFM images were recorded using the intermittent contact mode and silicon probes with a 10 nm tip radius, 240 kHz resonant frequency and 11 N/m force constant [21].

### Measurement of permeability with crystal violet

Alterations in the cell permeability were evaluated by crystal violet assay [22]. In brief, a culture of *S. aureus* 29213 was centrifuged, and the cells were resuspended in 0.5 mM potassium phosphate buffer, pH 7.4. The cells were incubated at 37 °C for 120 min with CTAB (0.3, 30, and 120 μg/mL) or with the 11FO d fraction at different MIC values ​​(1/2 to 8X). CTAB is a cationic detergent that increases cell permeability and therefore was used as a positive control. The negative control consisted of cells incubated only in PBS. The samples were centrifuged at 9300 x g for 5 min, resuspended in PBS containing 10 μg/mL of crystal violet and incubated at 37 °C for 10 min. After further centrifugation, the supernatants were collected and read at an OD of 590 nm. The absorbance of the violet crystal solution was also checked. The percentage of crystal violet uptake was calculated as the (OD of samples / OD of crystal violet solution) × 100.

### In vivo antimicrobial activity

*Galleria mellonella* was reared on an artificial diet at 25 °C. The diet consisted of a mix of 400 g wheat bran, 200 g wheat germ, 120 g dried beer yeast, 80 mL glycerin, 200 g skim milk powder, and 300 mL liquid honey. Larvae weighing 250–350 mg were divided into six groups. Unless otherwise stated, all the experiments involved groups containing 10 larvae. Group 1 was injected with a suspension of *S. aureus* ATCC 29213 at a 10^5^ CFU concentration. Group 2 received the same inoculum as Group 1 but 2 h after bacterial injection 10 μL of the 11FO d fraction were injected into larvae’s last proleg. Before injection, this fraction was diluted in PBS at a dose corresponding to 200 mg/kg. Every 24 h, a new dose of the fraction was applied, and the larvae were monitored for 96 h after the last application. A total of four doses of the fraction were applied to each larva in this group. Group 3 resembled Group 2, but instead, the 11FO d fraction was replaced by gentamicin at 200 mg/kg (G1397, Sigma-Aldrich, Dorset, UK). The other groups (4–6) consisted of uninfected controls, which were treated with a single injection containing 10 μL of PBS, the 11FO d fraction, or DMSO. The larvae were kept in petri dishes in a dark environment at 37 °C. The experiments were performed using three biological replicates.

### Statistical analyses

Survival data were collected using the Kaplan-Meier method, and the comparison between the control group and the treatment group was performed using the Log-rank test. A *p* ≤ 0.05 was considered significant. Data obtained from the agar well diffusion test and the cell permeability assay were subjected to one-way analysis of variance (ANOVA) followed by Dunnett’s test for comparison of more than two groups using PRISM 6 statistical software (San Diego, CA, USA). The results were considered statistically significant when *p* ≤ 0.05.

## Results

### Screening of extracts for antimicrobial activity

Twenty-six organic and aqueous extracts tested on *S. aureus* ATCC 29213 by the well diffusion test exhibited antimicrobial activity (Table 1). The largest inhibition halo was recorded for the organic extract of *Miconia petropolitana* leaves (24FO). The extracts from *Anadenanthera peregrina* led to the formation of inhibition zones ranging from 9 to 13 mm. Inhibition halos were observed when the leaf extracts from *Maclura tinctoria* (11FA and 11FO) were tested. Different extracts from *Bathysa nicholsonii*, *Piptadenia gonoacantha*, *Tabernaemontana hystrix*, *Xylopia sericea,* and *Myrciaria glazioviana* also showed activity against *S. aureus*. One extract from *Luehea grandiflora*, *Ceiba speciosa*, *Siparuna guianensis,* and *Alchornea glandulosa* produced inhibition halos. MIC assays were performed with all the extracts that resulted in inhibition zones and values ranged from 0.08 to 10 mg/mL (Table 1). The 11FO organic extract prepared from *Maclura tinctoria* leaves had the smallest MIC, and therefore, it was chosen for later assays.

### Phytochemical prospecting and bioactivity-guided fractionation of *Maclura tinctoria*

**Fig. 1:**
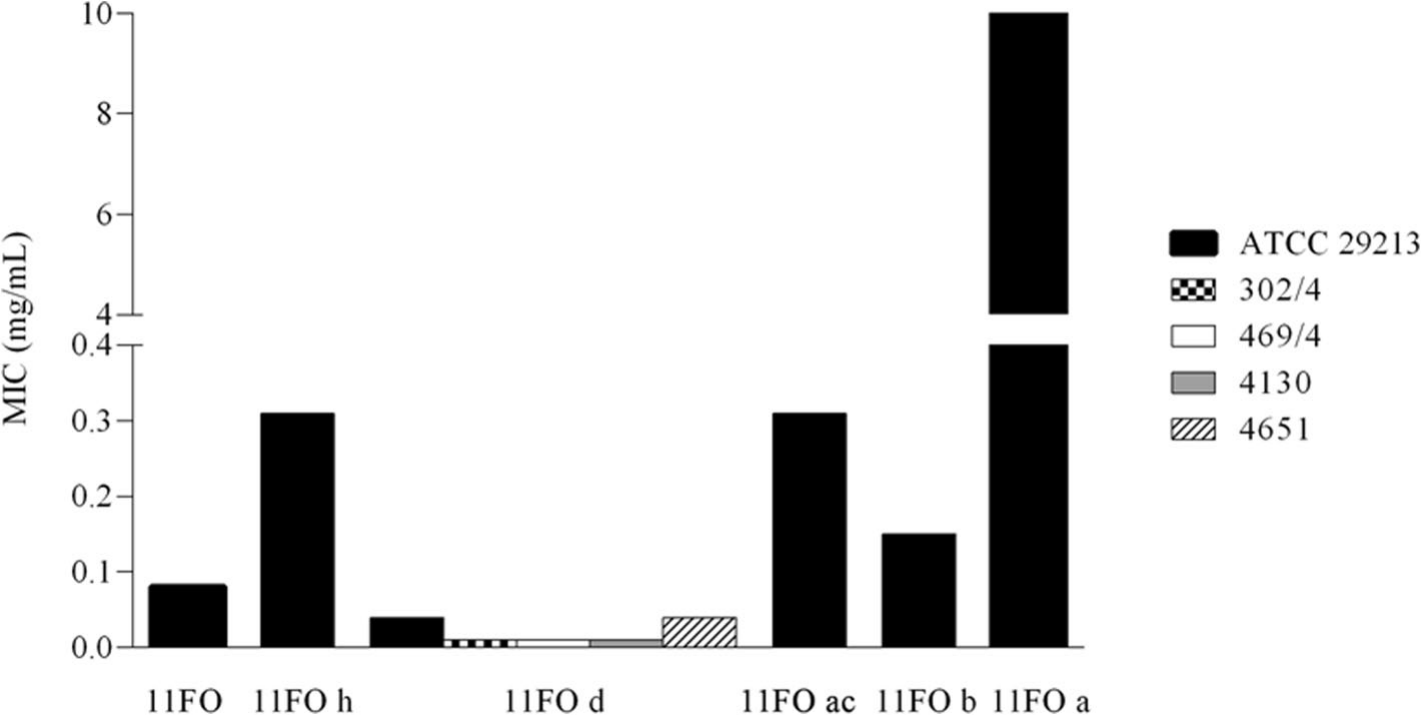
Determination of MICs for the 11FO fractions. 11FO h (hexane), 11FO d (dichloromethane), 11FO ac (ethyl acetate), 11FO b (n-butanol), and 11FO a (water) were evaluated on *Staphylococcus aureus* ATCC 29213, 302/4, 469/4, 4130, and 4651

Coumarins, flavonoids, essential oils, saponins, tannins and triterpenes/steroids were detected in the 11FO extract. The MICs of the fractions obtained with the solvents hexane (11FO h), ethyl acetate (11FO ac), n-butanol (11FO b), and water (11FO a) ranged from 0.04 to 10 mg/mL. The MIC obtained for the dichloromethane fraction of 11FO d was reduced to half that obtained for the crude extract (Fig. 1). The MIC of fraction 11FO d for bovine *S. aureus* isolates was also determined, and an antimicrobial activity between 0.01 and 0.04 mg/mL was recorded (Fig. 1). TLC showed that the fraction 11FO d consisted of a mixture of flavonoids.

**Fig. 2:**
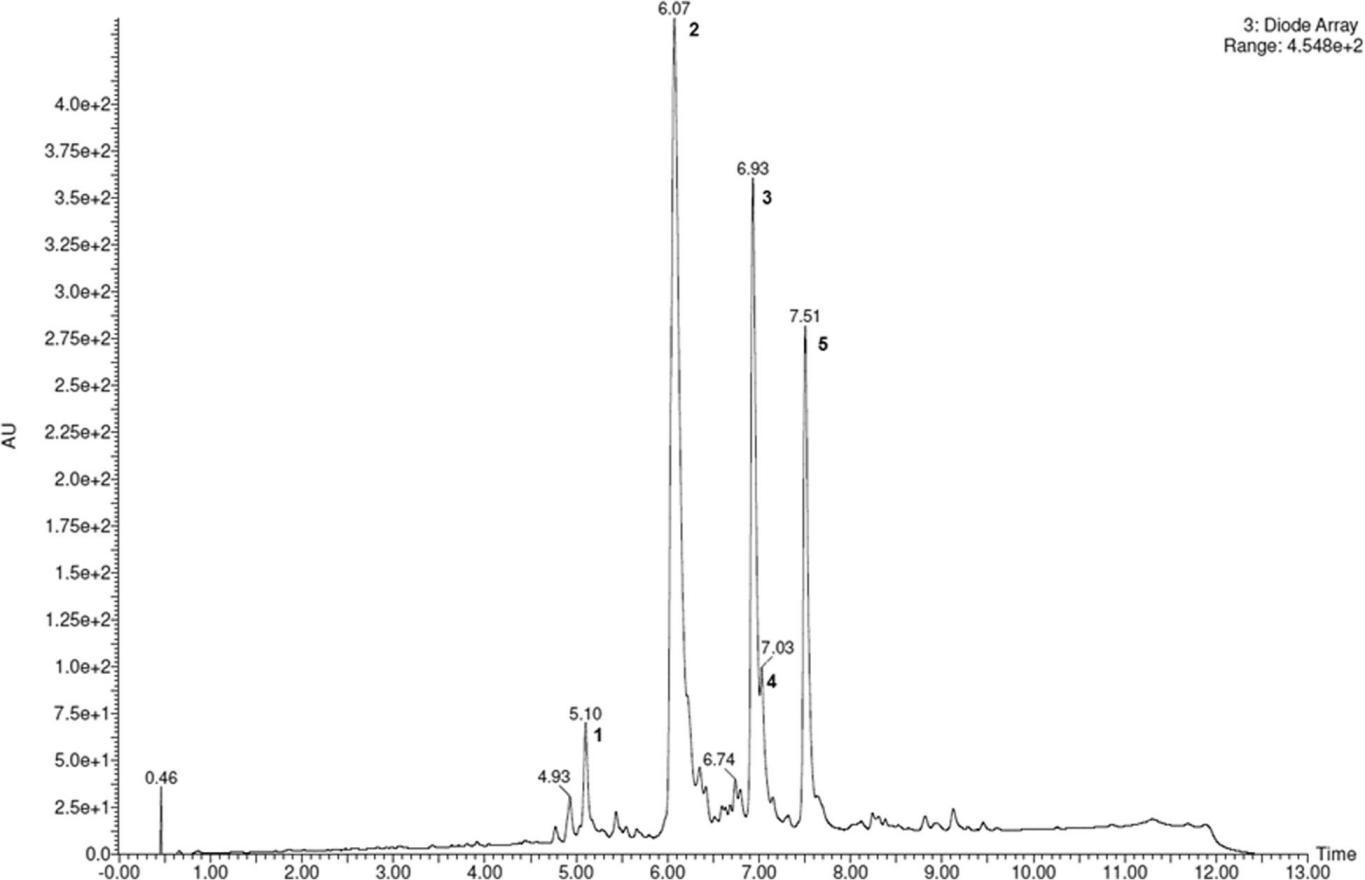
LC-DAD chromatogram of 11FO d from *Maclura tinctoria* as recorded at 270 nm

**Fig. 3:**
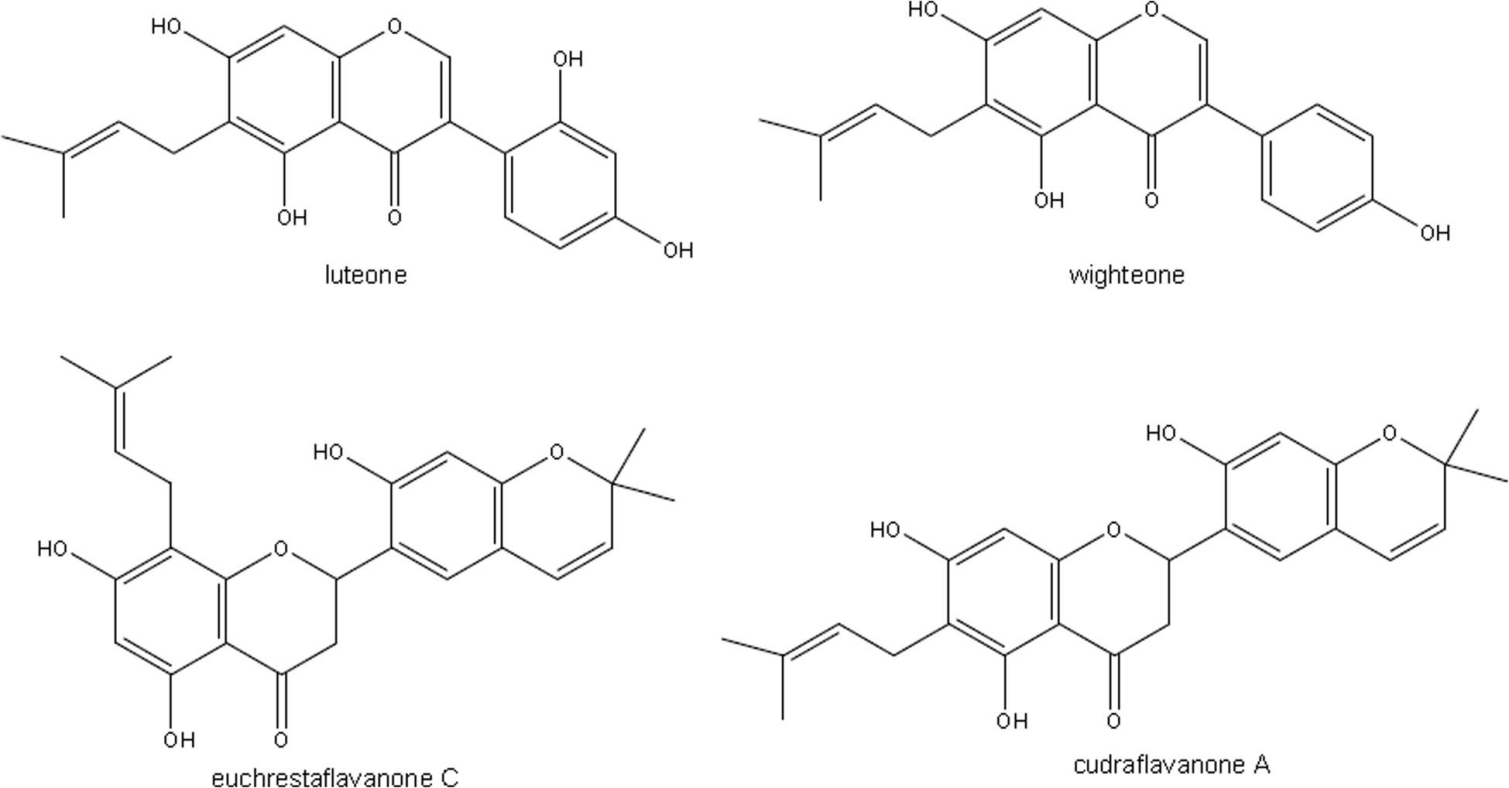
Prenylated flavonoids identified in the 11FO d fraction from *Maclura tinctoria.* The compounds were identified as luteone (1), wightenone (2), euchrestaflavanone C (3), cudraflavanone A (4), and diprenylated flavanone

The phytochemical profile of active fraction 11FO d was determined by UPLC-DAD (Fig. 2), which showed the presence of five major compounds. All compounds showed UV spectra with maximum absorption (λ_max._) near 264 and 270 nm, which are typical wavelength values for isoflavone or flavanone derivatives. Full scan mass spectra (100–1500 Da) were obtained for 11FO d by LC-DAD-ESI-MS in positive and negative modes, and their molecular ion and fragmentation analyses allowed us to confirm the structures of five prenylated flavonoids (Fig. 3). Compound **1** showed the MS spectrum in negative mode presented an [M-H]^−^ at *m/z* 353. Positive mode MS^2^ focused on *m/z* 355, *m/z* 337 [M + H-18]^+^ and *m/z* 299, which could be attributed due to the loss of a prenyl unit (56 u). Data analysis along with literature reports on the Moraceae family suggests that compound **1** is luteone. Compound **2** was the major constituent of 11FO d and presented an [M + H]^+^ ion at *m/z* 339 with an abundant product ion at *m/z* 283 that was attributed to a loss of 56 Da, which is characteristic of isoflavone compounds. The molecular formula of this ion (C_20_H_18_0_5_) and its fragmentation and absorption maximum (λ_max._) at 266 suggest that this compound is the prenylated isoflavone known as wightenone. Spectra obtained for peaks **3** and **4** showed the UV absorption maxima with two bands at 270 nm, which are characteristic of the flavanone derivatives. The ESI-MS for both compounds showed the same [M + H]^+^-protonated molecule at *m/z* 423 and [M-H]^−^-deprotonated molecule at *m/z* 421 with very similar retention times, suggesting the presence of isomers. These protonated molecules refer to isomers of flavanones that are attached to two prenyl substituents on their aglycone. In the negative mode, compound 3 presented fragmentation ions at *m/z* 367 which indicates the loss of a ring-closed prenyl group, C_4_H_6_. The fragment at *m/z* 293 [M-H-54-56-18] indicates loss of a second neutral prenyl group, C_4_H_8_, plus a water molecule. These data indicated that peaks **3** and **4** refer to isomers of flavanones with two prenyl substituents, one in an open chain form and the other in a closed ring form, confirming the presence of euchrestaflavanone C and cudraflavanone A (C_25_H_26_O_6_). Compound **5** also showed spectra suggestive of a flavanone (λ_max_ 270 nm). The MS spectrum in positive mode was focused on a m/*z* 407 with an abundant product ion at *m/z* 351 that was attributed to a loss of 56 Da, which is characteristic of the presence of prenyl group. The spectral data suggest that compound **5** is a diprenylated flavanone.

### Effect of fraction 11FO d on *S. aureus* ATCC 29213

**Fig. 4:**
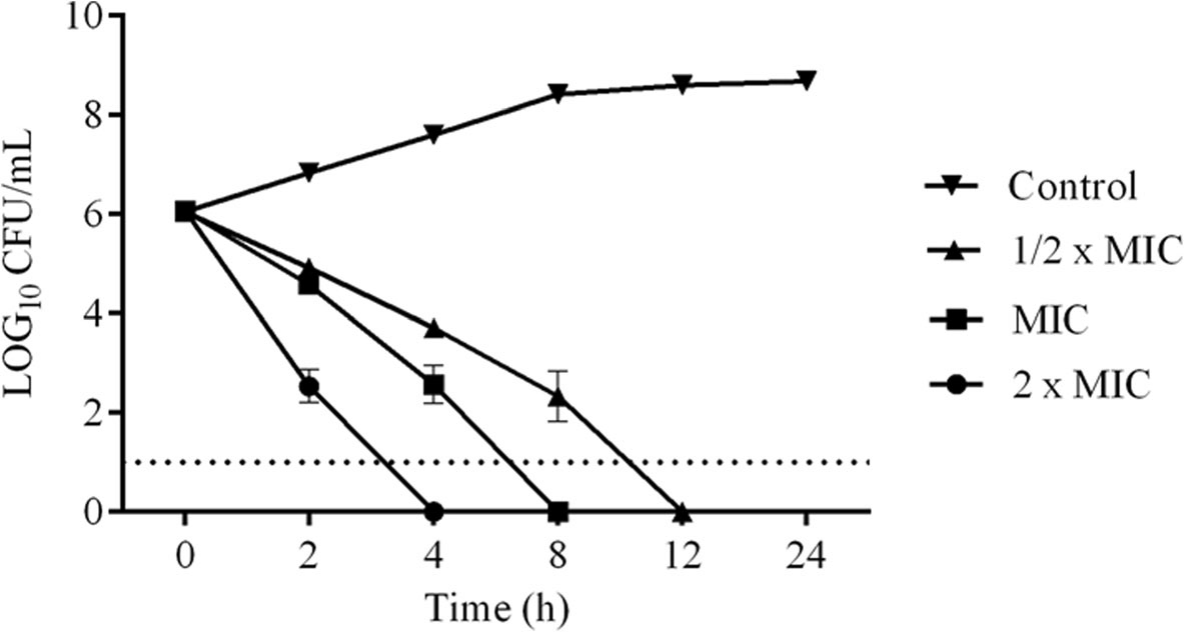
Effect of different inhibitory concentrations of the 11FO d fraction on *Staphylococcus aureus* ATCC 29213

**Fig. 5:**
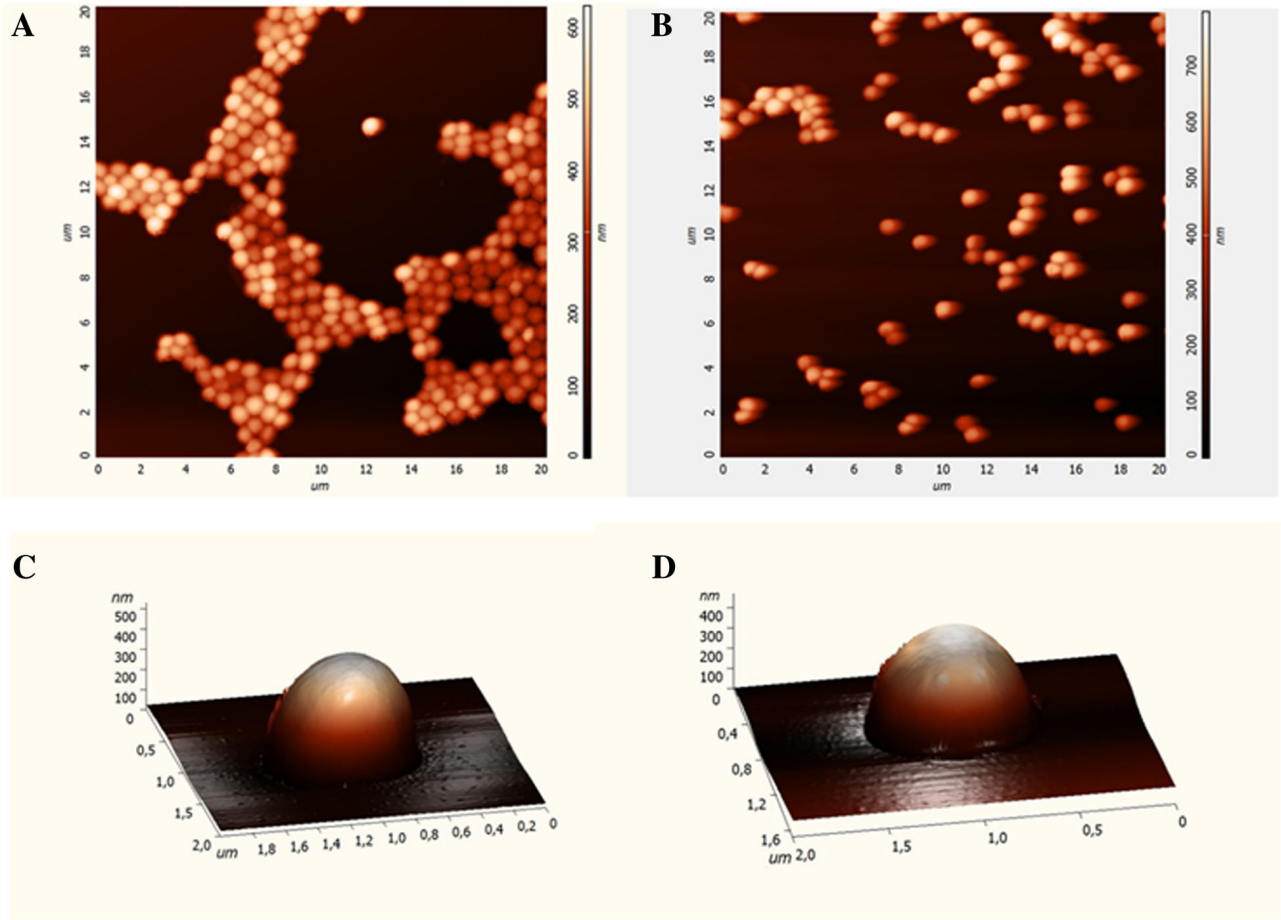
Atomic force microscopy of *Staphylococcus aureus* ATCC 29213 cells exposed (**b** and **d**) and not exposed (**a** and **c**) to fraction 11FO d for 4 h at 37 °C

**Fig. 6:**
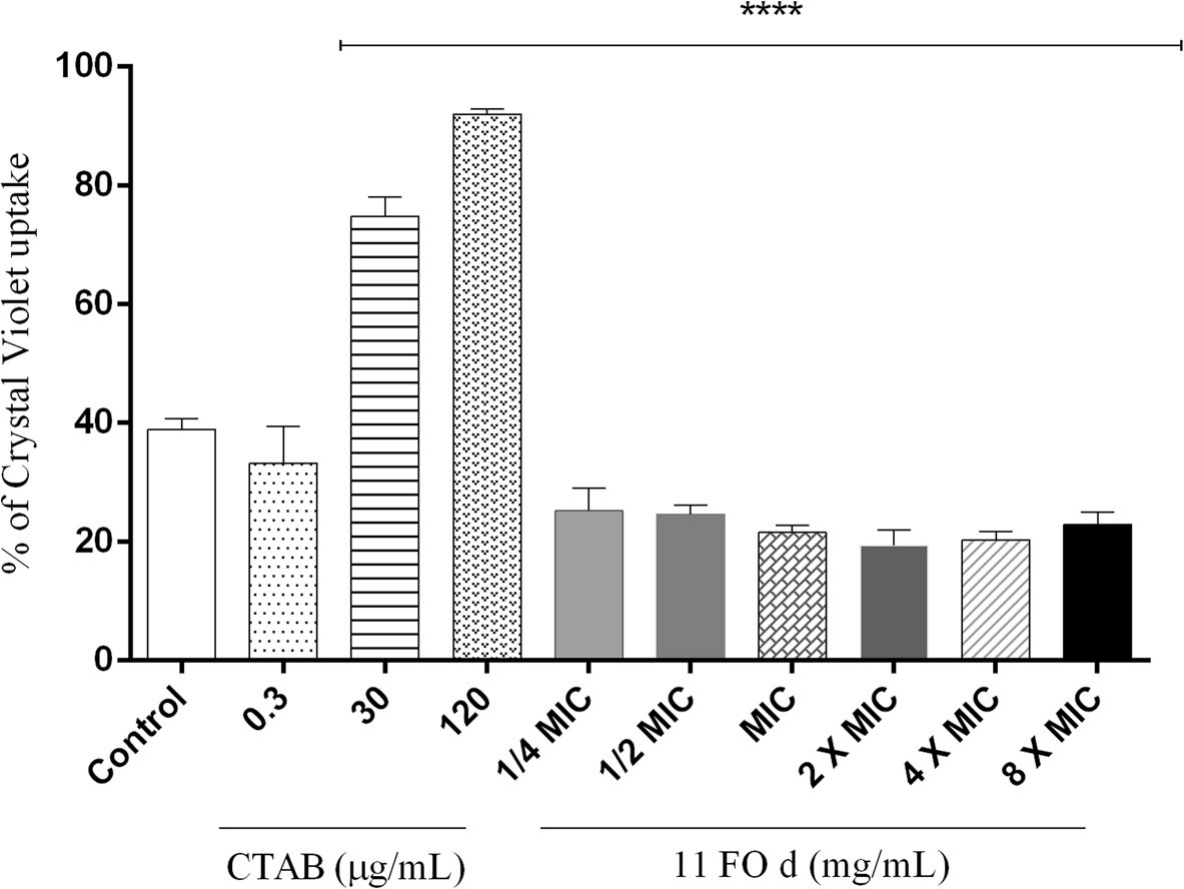
Measurement of cell permeability. Bacterial cells were incubated in PBS (control) or in PBS containing different concentrations of CTAB or 11FO d and were then exposed to 10 μg/mL crystal violet for 10 min. The culture supernatant was collected for an absorbance reading at 590 nm. Means obtained for three biological replicates. ****p < 0.0001, significantly different from the control (PBS)

After 4, 8, and 12 h of exposure to 2X MIC, MIC and 1/2 MIC, there was no further bacterial growth, which revealed the bactericidal effect of the fraction (Fig. 4). To explore the possible antibacterial mechanism of fraction 11FO d, the morphological changes in *S. aureus* were observed by AFM. No remarkable changes in the bacterial surface or cellular dimensions (1 μm × 500 nm) were apparent, although a decrease in the number of bacteria was observed after 4 h of treatment, as were changes in the cellular arrangement (Fig. 5). The alterations in cellular permeability were tested by incubating the cells with crystal violet, a hydrophobic dye that is capable of crossing the lipid bilayer (Fig. 6). In the presence of increasing concentrations of CTAB, a cationic detergent, there was greater dye entry, which is consistent with the increased permeability of cell membrane. The same trend was not observed when the cells were incubated in the presence of the 11FO d fraction, suggesting that the membrane properties were not altered by the compounds found in the fraction. Cells treated with the fraction had a lower uptake of crystal violet in relation to the cells without the added fraction (control).

### In vivo antimicrobial activity

**Fig. 7:**
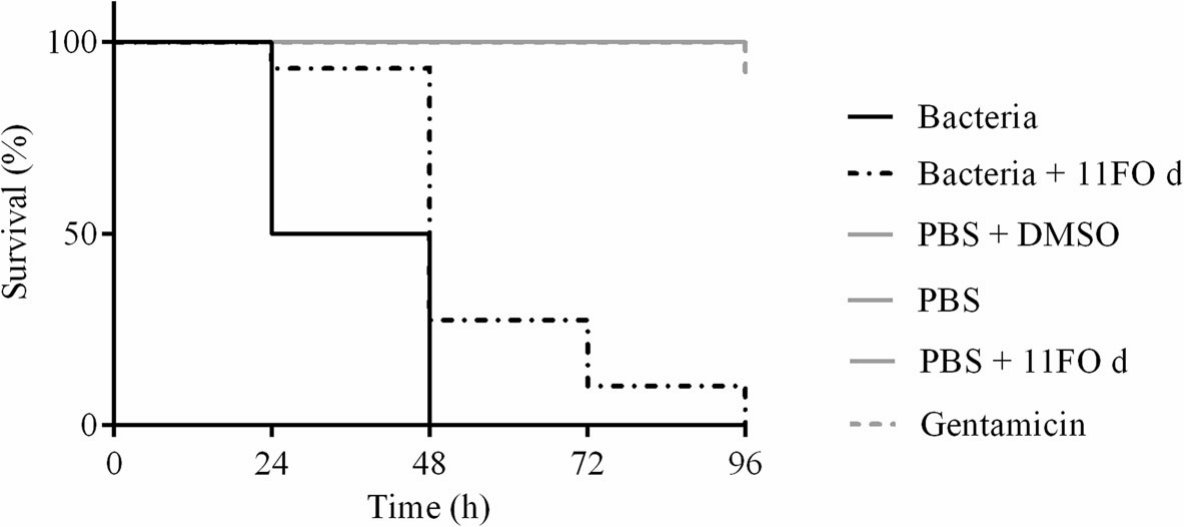
Survival of *Galleria mellonella* inoculated with *Staphylococcus aureus* ATCC 29213. Larvae were treated with 200 mg/kg 11FO d at 96 h after infection. ****p < 0.0001, significantly different from the control (PBS)

The 11FO d fraction used at the 200 mg/kg concentration increased the survival of insects that were infected with *S. aureus* ATCC 29213 when compared to those in the control groups. After 24 h, 50 % of the larvae were dead compared to the high survival seen in the group that received the fraction. After 48 h, there were still live larvae in the group treated with 11FO d. All the larvae injected with PBS, PBS and DMSO, or PBS and extract survived after 96 h of monitoring (Fig. 7).

## Discussion

The relevance of the Atlantic Forest biome as a reservoir of chemical compounds with antibiotic potential was demonstrated in this work. Of the 23 species tested, 12 presented antimicrobial activity against *S. aureus,* with highlights for *Tabernaemontana hystrix*, *Anadenanthera peregrina*, *Miconia petropolitana*, *Xylopia sericea*, *Myrciaria glazioviana,* and *Maclura tinctoria*. This is the first report of the antibacterial activity of species such as *T. hystrix*, which has been described only for its anticholinesterase activity, [23] and *A. peregrina*, for its antioxidant property [24]. No studies on *M. petropolitana* have been found, although other species of this genus are known for their antiparasitic, antibiotic, analgesic, and antitumor activities [25].

Among all species evaluated in the present study, *M. tinctoria* showed the most promising antimicrobial activity. *M. tinctoria* D. Don Ex Steud is a tree species of the family Moraceae, with wide distribution in Brazil. The bioassay-guided fractionation conducted here resulted in a fraction enriched in prenylated isoflavones and flavanones, which led to a greater reduction in MIC compared to the crude extract, evidencing that these compounds are responsible for the antibacterial activity seen. The dichloromethane fraction (11FO d) also showed a bactericidal action, which contributes to a faster elimination of the pathogen, decreasing the probability of infection propagation and the emergence of resistance when compared to a bacteriostat. Prenylated flavanones and isoflavones are flavonoids that have already been identified in the *Maclura* genus [26, 27]. Although there is already a report on the antibacterial activity of extracts from *M. tinctoria* [28], the present work corroborates this result with new scientific evidence, also associating the secondary metabolites responsible for this activity to this species.

Studies performed with plant extracts are usually restricted to in vitro assay methods. Here, the in vivo model indicated that the fraction 11FO d conferred a remarkable degree of protection against *S. aureus* ATCC 29213. Additionally, this model demonstrated that the experimental dose used (200 mg/kg) was not toxic to the larvae. Further studies are underway to investigate the possible synergism of fraction 11FO d with commonly used antibiotics and the effect of other dose regimens. The use of *G. mellonella* to evaluate the efficacy of phytochemicals has been increasing in recent years [29], and the results show that the larvae are a good alternative for assessing the in vivo efficacy of plant extracts, although tests in standard models should be conducted. Furthermore, larvae rearing is quite inexpensive and experimentation does not require ethical approval [30].

The antibacterial compounds of *M. tinctoria* had no effect on the cell surface since the *S. aureus* cells treated with the fraction 11FO d had similar morphology to the control but lost typical grape-like clusters arrangement. Fraction 11FO is rich in prenylated flavanones and isoflavones, which are hydrophobic compounds that could, in principle, disrupt the membrane function. However, no change in cell membrane permeability was detected by the method used herein. Since prenylation can contribute to diversification of flavonoids [31] the strong antibacterial activity seen may result from the interaction of the compounds with different bacterial targets.

## Conclusion

This is the first report of the antibacterial activity of *T. hystrix* and *A. peregrine*. Flavanones and isoflavones were enriched in the active fraction of *M. tinctoria* against *S. aureus* that protected infected larvae suggesting a possible use of prenylated flavanones and isoflavones as antistaphylococcal agents. Further research is ongoing to elucidate the mechanisms of action of these compounds and to evaluate their synergistic effects with other antimicrobials.

## Data Availability

The datasets used and/or analyzed during the current study available from the corresponding author on reasonable request.

## Abbreviations

AFM: Atomic force microscopy
ANOVA: Analysis of variance
BHI: Brain heart infusion
CA-MRSA: Community-acquired infections
CFU: Colony forming units
CLSI: Clinical and Laboratory Standards Institute
CTAB: Cetyl trimethylammonium bromide
DMSO: Dimethyl sulfoxide
HA- MRSA: Community-acquired infections
LC-DAD-ESI/MS: Liquid chromatography with diode array detection and electrospray ionization mass spectrometry
MH: Müeller-Hinton
MIC: Minimum inhibitory concentration
MRSA: Methicillin-resistant *Staphylococcus aureus*
PBS: Phosphate-buffered saline

## References

[1] Kobayashi SD, Malachowa N, DeLeo FR (2015). Pathogenesis of *Staphylococcus aureus* abscesses. Am J Pathol.

[2] Peton V, Le Loir Y (2014). *Staphylococcus aureus* in veterinary medicine. Infect Genet Evol.

[3] Chambers HF, DeLeo FR (2009). Waves of resistance: *Staphylococcus aureus* in the antibiotic era. Nat Rev Microbiol.

[4] Otto M (2013). Community-associated MRSA: what makes them special?. Int J Med Microbiol.

[5] Hammer KA, Carson CF, Riley TV (1999). Antimicrobial activity of essential oils and other plant extracts. J Appl Microbiol.

[6] Kumari R, Kumar S, Kumar A, Goel KK, Dubey RC (2017). Antibacterial, antioxidant and immuno-modulatory properties in extracts of *Barleria lupulina* Lindl. BMC Complement Altern Med.

[7] Li B, Pai R, Di M, Aiello D, Barnes MH, Butler MM, Tashjian TF, Peet NP, Bowlin TL, Moir DT (2012). Coumarin-based inhibitors of *Bacillus anthracis* and *Staphylococcus aureus* replicative DNA helicase: chemical optimization, biological evaluation, and antibacterial activities. J Med Chem.

[8] Hou Z, Zhou Y, Li J, Zhang X, Shi X, Xue X, Li Z, Ma B, Wang Y, Luo X (2015). Selective *in vivo* and *in vitro* activities of 3, 3′-4-nitrobenzylidene-bis-4-hydroxycoumarin against methicillin-resistant *Staphylococcus aureus* by inhibition of DNA polymerase III. Sci Rep.

[9] Teng Z, Shi D, Liu H, Shen Z, Zha Y, Li W, Deng X, Wang J (2017). Lysionotin attenuates *Staphylococcus aureus* pathogenicity by inhibiting α-toxin expression. Appl Microbiol Biotechnol.

[10] McCloud TG (2010). High throughput extraction of plant, marine and fungal specimens for preservation of biologically active molecules. Molecules.

[11] CLSI. Methods for Dilution Antimicrobial Susceptibility Tests for Bacteria That Grow Aerobically; Approved Standard-Ninth Edition. CLSI document M7-A9. Wayne: Clinical and Laboratory Standards Institute; 2012.

[12] Wagner H, Bladt S, Zgainski EM (1984). Plant drug analysis. A thin layer chromatography atlas.

[13] Gontijo DC, Brandão GC, Gontijo PC, Oliveira AB, Diaz MAN, Fietto LG, Leite JPV (2017). Identification of phenolic compounds and biologically related activities from *Ocotea odorifera*aqueous extract leaves. Food Chem.

[14] Lin LZ, Harnly JM (2012). Quantitation of flavanols, proanthocyanidins, isoflavones, flavanones, dihydrochalcones, stilbenes, benzoic acid derivatives using ultraviolet absorbance after identification by liquid chromatography–mass spectrometry. J Agric Food Chem.

[15] Simons R, Vincken JP, Bakx EJ, Verbruggen MA, Gruppen H (2009). A rapid screening method for prenylated flavonoids with ultra-high-performance liquid chromatography/electrospray ionization mass spectrometry in licorice root extracts. Rapid Commun Mass Spectrom.

[16] Xu MJ, Wu B, Ding T, Chu JH, Li CY, Zhang J, Wu T, Wu J, Liu SJ, Liu SL, Ju WJ, Li P (2012). Simultaneous characterization of prenylated flavonoids and isoflavonoids in *Psoralea corylifolia* L. by liquid chromatography with diode-array detection and quadrupole time-of-flight mass spectrometry. Rapid Commun Mass Spectrom.

[17] Fongang YSF, Bankeu JJK, Ali MS, Awantu AF, Zeeshan A, Assob CN, Mehreen L, Lenta BN, Ngouela SA, Tsamo E (2015). Flavonoids and other bioactive constituents from *Ficus thonningii* Blume (Moraceae). Phytochem Lett.

[18] Oyama SO, de Souza LA, Baldoqui DC, Sarragiotto MH, Silva AA (2013). Prenylated flavonoids from *Maclura tinctoria* fruits. Quím Nova.

[19] Han HJ, Kim TJ, Jin YR, Hong SS, Hwang JH, Hwang BY, Lee KH, Park TK, Yun YP (2007). Cudraflavanone A, a flavonoid isolated from the root bark of *Cudrania tricuspidata*, inhibits vascular smooth muscle cell growth via an Akt-dependent pathway. Planta Med.

[20] Li J, Dong J, Qiu JZ, Wang JF, Luo MJ, Li HE, Leng BF, Ren WZ, Deng XM (2011). Peppermint oil decreases the production of virulence-associated exoproteins by *Staphylococcus aureus*. Molecules.

[21] Santana HF, Barbosa AAT, Ferreira SO, Mantovani HC (2012). Bactericidal activity of ethanolic extracts of propolis against *Staphylococcus aureus* isolated from mastitic cows. World J Microbiol Biotechnol.

[22] Halder S, Yadav KK, Sarkar R, Mukherjee S, Saha P, Haldar S, Karmakar S, Sen T (2015). Alteration of Zeta potential and membrane permeability in bacteria: a study with cationic agents. Springerplus.

[23] Marinho FF, Simões AO, Barcellos T, Moura S (2016). Brazilian *Tabernaemontana* genus: indole alkaloids and phytochemical activities. Fitoterapia.

[24] Mensor LL, Menezes FS, Leitão GG, Reis AS, Santos TCD, Coube CS, Leitão SG (2001). Screening of brazilian plant extracts for antioxidant activity by the use of DPPH free radical method. Phytother Res.

[25] Cunha WR, Martins C, da Silva FD, Crotti AEM, Lopes NP, Albuquerque S (2003). *In vitro* trypanocidal activity of triterpenes from *Miconia* species. Planta Med.

[26] Lee SJ, Wood AR, Maier MGA, Dixon RA, Mabry TJ (1998). Prenylated flavonoids from *Maclura pomifera*. Phytochemistry..

[27] El-Sohly HN, Joshi A, Li XC, Ross SA (1999). Flavonoids from *Maclura tinctoria*. Phytochemistry.

[28] Alvaro MR, Alejandra HH, Antonio DC (2015). *In vitro* antibacterial activity of *Maclura tinctoria* and *Azadirachta indica* against *Streptococcus mutans* and *Porphyromonas gingivalis*. Br J Pharm Res.

[29] Ferro TA, Araújo JM, dos Santos PBL, dos Santos JS, Souza EB, da Silva BL, Colares VLP, Novais TMG, Struve C, Calixto JB, Monteiro-Neto V, da Silva LCC, Fernandes ES (2016). *Cinnamaldehyde* inhibits *Staphylococcus aureus* virulence factors and protects against infection in a *Galleria mellonella* model. Front Microbiol.

[30] Tsai CJ-Y, Loh JMS, Proft T (2016). *Galleria mellonella* infection models for the study of bacterial diseases and for antimicrobial drug testing. Virulence.

[31] Yazaki K, Sasaki K, Tsurumaru Y (2009). Prenylation of aromatic compounds, a key diversification of plant secondary metabolites. Phytochemistry.

